# Bilateral Central Retinal Vein Occlusion in Recurrent Frontal Lobe Tumor

**DOI:** 10.7759/cureus.41441

**Published:** 2023-07-06

**Authors:** Priyadarshini Mishra, Shanmugasundaram P, Bijnya B Panda, Arunkumar Sekar, Suprava Naik

**Affiliations:** 1 Ophthalmology, All India Institute of Medical Sciences, Bhubaneswar, Bhubaneswar, IND; 2 Neurosurgery, All India Institute of Medical Sciences, Bhubaneswar, Bhubaneswar, IND; 3 Radiodiagnosis, All India Institute of Medical Sciences, Bhubaneswar, Bhubaneswar, IND

**Keywords:** hypercoagulable state, brain tumor, systemic malignancy, retinal vein occlusion, central retinal vein occlusion

## Abstract

Systemic malignancy can induce hypercoagulation and can cause retinal vein occlusion (RVO). Although RVO has been reported in association with breast, renal, lung, prostate, and ovarian malignancies, it has not been reported in brain tumors. We are reporting a case of bilateral central retinal vein occlusion (CRVO) associated with recurrent frontal lobe gliosarcoma. The association was established after ruling out all other systemic causes that can produce bilateral CRVO. The importance of this case report lies in the fact that, while evaluating bilateral CRVO cases, these rare associations should also be kept in mind.

## Introduction

Central retinal vein occlusion (CRVO) is one of the most common acquired retinal vascular diseases in the older population and is found in 0.8 per 1000 persons [1]. CRVO can manifest as two distinct entities, ischemic and non-ischemic varieties [2]. The pathogenesis of CRVO is due to thrombotic occlusion of the central retinal vein (CRV) at or posterior to the lamina cribrosa [3]. Advanced age, systemic hypertension, hyperlipidemia, and hyperglycemia are important risk factors for this entity. Other conditions such as papilledema, glaucoma, and optic nerve head drusen can also predispose the patient to CRVO [3,4]. In young patients, retinal vasculitis, hypercoagulable states, and primary open-angle glaucoma are usually associated [5].

Systemic malignancies can have ocular manifestations such as metastatic tumors and carcinoma-associated retinopathy or can induce a hypercoagulable state, causing retinal vein occlusion (RVO). Although RVO has been reported in association with breast, renal, lung, prostate, and ovarian malignancies [6-10], there is no report regarding intracranial malignancy causing this condition. Herein, we report a case of bilateral CRVO in a patient with recurrent frontal lobe gliosarcoma. The complex pathophysiology with multiple suspected causal factors makes this case unique.

## Case presentation

A 40-year-old male came to our outpatient department for decreased vision in both eyes. He was referred from the Department of Neurosurgery with a diagnosis of recurrent frontal lobe gliosarcoma. He had no history of diabetes, hypertension, dyslipidemia, or recent COVID infection or vaccination.

**Figure 1:**
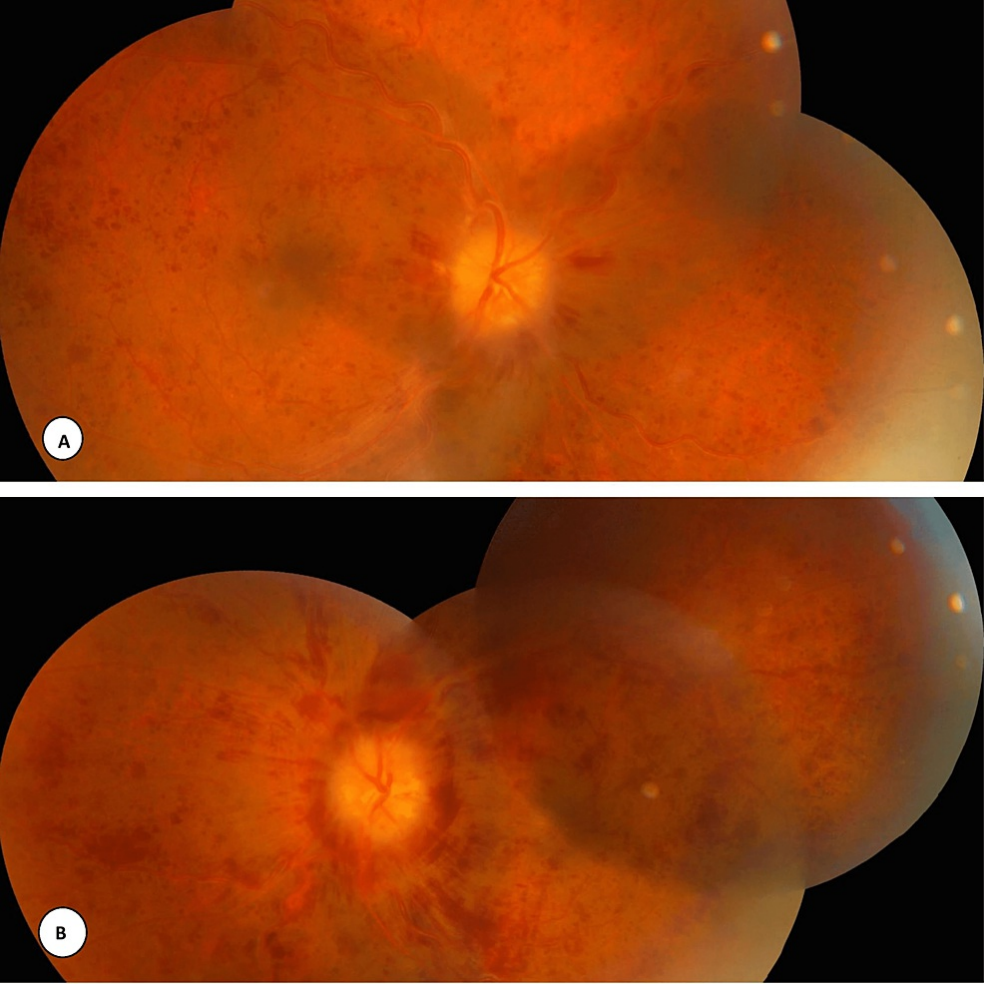
Fundus photo: A) right eye and B) left eye The fundus picture (montage) of (A: right eye, B: left eye) revealed optic disc edema with mild pallor, venous dilatation and tortuosity, peripapillary hemorrhages, and extensive superficial hemorrhages in all quadrants.

A thorough ocular examination was done. Visual acuity in the right eye was 1/60 and, in the left eye, finger counting close to the face. No ocular motility defect was noticed. Intraocular pressure in both eyes was 15 mm Hg by non-contact tonometry. The pupil was sluggishly reacting to light. On slit-lamp examination, the anterior segment in both eyes was normal. Fundus examination revealed optic disc edema with pallor, venous dilatation and tortuosity, and extensive superficial hemorrhage in all quadrants suggestive of bilateral CRVO (Figure 1).

The blood pressure recorded was 130/84 mm Hg.

**Figure 2:**
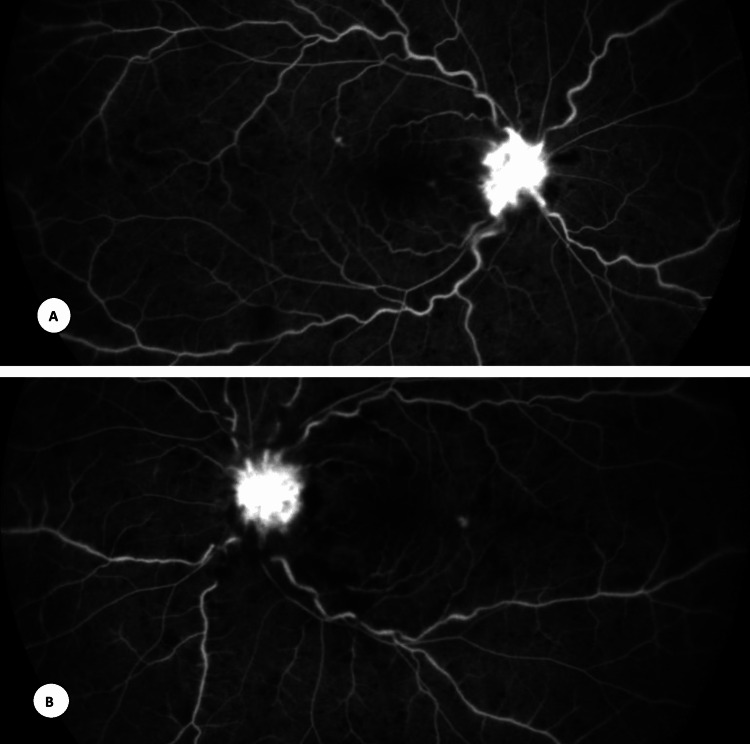
Fundus fluorescein angiography: A) right eye and B) left eye Fundus fluorescein angiography late phase (A: right eye, B: left eye) showing leakage from the disc, a blocked effect due to hemorrhages. No capillary nonperfusion areas were seen.

On fundus fluorescein angiography, there was leakage from the disc, a blocked effect due to hemorrhages. It was confirmed to be a bilateral non-ischemic type of CRVO (Figure 2).

**Figure 3:**
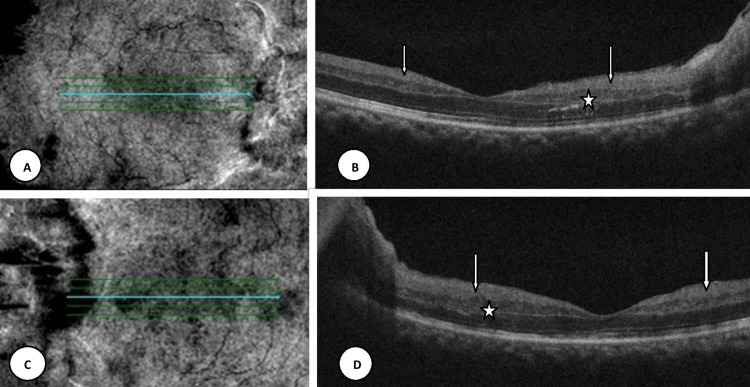
SD-OCT scan of the macula Near-infrared reflectance image and OCT scan (HD raster macula) of the right eye (A,B) and left eye (C,D), showing thickening and disorganization of the nerve fiber layer, ganglion cell layer, and inner plexiform layer (white arrows). Disorganization of the inner nuclear and outer plexiform layer also seen with focal extension into the outer nuclear layer (asterisk).

An optical coherence tomography (OCT) scan of the macula showed thickening and disorganization of the inner and middle layers of the retina (Figure 3).

Five main possibilities were kept in mind regarding the pathophysiology of bilateral CRVO in this patient: first, CRVO due to systemic risk factors not associated with tumors; second, CRVO due to papilledema; third, direct microinvasion of tumor causing venous obstruction; fourth, malignancy-induced hypercoagulation; and, fifth, drug-induced aplastic anemia causing papilledema along with hemorrhagic retinopathy. 

Extensive systemic investigations were done. Complete blood count, peripheral smear, blood sugar, lipid profile, renal function test, serum homocysteine, C-reactive protein, serum electrophoresis, protein C, protein S, serum angiotensin-converting enzyme, chest X-ray, HBsAg, HCV, HIV screening all came out to be normal. Autoimmune workup and COVID antibody were negative. The blood coagulation profile showed only a mildly elevated fibrinogen level (560 mg/dl). Bleeding time, clotting time, and prothrombin time/international normalized ratio (PT-INR) were within normal limits.

Based on all these investigations, the possibility of CRVO due to any other systemic cause was ruled out. A hypercoagulation mechanism also could not be proved based on only increased fibrinogen. Hemorrhagic retinopathy due to anemia was excluded as blood counts were within normal limits.

**Figure 4:**
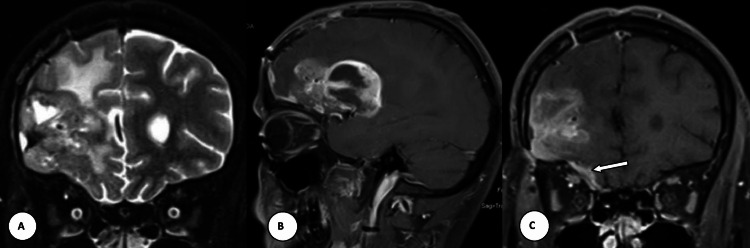
MRI image of the brain and orbit MRI of the brain and orbit. (A) Coronal T2WI shows an ill-defined heterogeneously hyperintense lesion in the right frontal lobe. The lesion is showing intense and heterogeneous enhancement in the sagittal (B) and coronal (C) post-contrast T1WI. Small intraorbital extraconal extension noted at the superomedial aspect of the right orbit (arrow in C).

MRI brain and orbit show an ill-defined heterogeneously hyperintense lesion in the right frontal lobe. The orbital apex was free without any obstruction. Small intraorbital extraconal extension was noted at the superomedial aspect of the right orbit although that cannot explain orbital tissue compression and venous stasis in both eyes (Figure 4).

**Figure 5:**
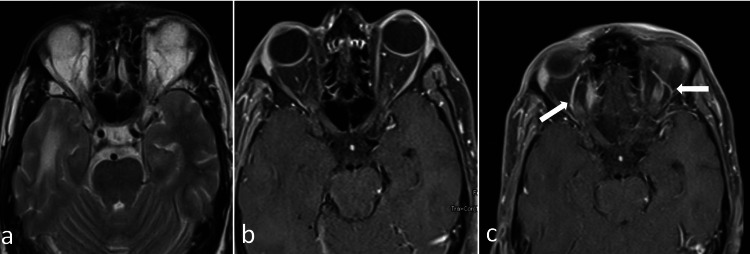
MRI of the brain and orbit MRI brain and orbit: axial T2WI (a) and post-contrast T1WI with fat suppression (b and c) shows a normal orbital apex. Post-contrast T1WI showing bilateral superior ophthalmic veins (arrows in c). Right side being mildly prominent than the left side.

Although, in MRI, superior ophthalmic veins were grossly normal on both sides, microinvasion by tumor could not be ruled out (Figure 5).

Hence, long-standing papilledema compressing the optic nerve and central retinal veins was the most probable cause of bilateral CRVO and decreased vision in this case. 

The patient underwent re-surgery, followed by fractionated focal irradiation with concomitant chemotherapy with temozolomide. On follow-up at two months, retinal hemorrhages were decreased but vision did not improve. 

## Discussion

Bilateral CRVO can be the first manifestation of a potentially life-threatening disease. It mandates a thorough systemic investigation to rule out all the possible causes of CRVO. If more common predisposing factors such as hyperglycemia, hypertension, hyperlipidemia, and smoking history are absent, and in particularly young patients, other causes such as vasculitis, hereditary, and acquired hypercoagulable states need to be investigated.

Our case presented a very complex scenario where multiple factors could have contributed toward the pathophysiology of bilateral CRVO. We carefully ruled out all possible systemic associations by thorough investigations.

Malignancy and myeloproliferative disorders are known causes to induce a hypercoagulation state and increase the risk of thromboembolic complications. The risk of venous thromboembolism (VTE) is high for patients with brain tumors, particularly during the first month after neurosurgery. Glioblastoma patients, in particular, have the highest risk of VTE [11-13]. In our case, we could not prove this mechanism. More detailed hematological investigations are necessary in such a scenario.

Temozolomide was used as chemotherapy in our case, which has been reported to cause aplastic anemia [14]. Aplastic anemia can cause raised intracranial pressure [15,16] and hemorrhagic retinopathy. As the total white blood cell and red blood cell count and hemoglobin level were within normal limits, hemorrhage due to anemia was ruled out. 

As gliosarcoma is highly invasive in nature [17], the possibility of direct microinvasion causing venous obstruction cannot be ruled out in this case, but local invasion could not explain the bilateral involvement.

The most probable causal factor in this case was long-standing papilledema, which compressed bilateral optic nerve and central retinal veins causing decreased vision and venous stasis retinopathy seen as widespread retinal hemorrhages. Although optic disc edema with peripapillary hemorrhages is the most common finding in papilledema, unusual hemorrhagic patterns such as prominent sub-hyaloid and vitreous hemorrhage and peripheral retinal hemorrhage have also been reported [18-20]. 

## Conclusions

A young male with recurrent frontal lobe gliosarcoma presented with decreased vision in both eyes. On examination, he was found to have optic disc swelling with mild pallor, widespread retinal hemorrhages in both eyes, and, on the investigation, mildly elevated fibrinogen level in the absence of any other systemic causes. The diagnosis was most probably bilateral CRVO and optic nerve compression due to long-standing papilledema. 

A thorough systemic investigation is mandatory in a complex situation such as our case where the possibility of multiple causal factors contributing toward the pathophysiology of bilateral CRVO is suspected. A detailed hematological workup should be done to confirm the association with malignancy-induced hypercoagulation. These rare associations should always be kept in mind while evaluating retinal vein occlusion cases. 

## References

[1] Rogers S, McIntosh RL, Cheung N (2010). The prevalence of retinal vein occlusion: Pooled data from population studies from the United States, Europe, Asia, and Australia. Ophthalmology.

[2] Hayreh SS (1983). Classification of central retinal vein occlusion. Ophthalmology.

[3] Green WR, Chan CC, Hutchins GM, Terry JM (1981). Central retinal vein occlusion: A prospective histopathologic study of 29 eyes in 28 cases. Retina.

[4] Koizumi H, Ferrara DC, Bruè C, Spaide RF (2007). Central retinal vein occlusion case-control study. Am J Ophthalmol.

[5] Chen TY, Uppuluri A, Zarbin MA, Bhagat N (2021). Risk factors for central retinal vein occlusion in young adults. Eur J Ophthalmol.

[6] Madanagopalan VG, Paneer Selvam V, Sarath Sivan NV, Govindaraju NV (2019). Central retinal vein occlusion in a patient with breast carcinoma. GMS Ophthalmol Cases.

[7] Adrean SD, Schwab IR (2003). Central retinal vein occlusion and renal cell carcinoma. Am J Ophthalmol.

[8] Castro-Navarro V, Odaibo SG, Ghodasra DH, Besirli CG (2015). Bilateral BRVO in a patient with recurrent prostate cancer. BMJ Case Rep.

[9] Ronchetto F (1994). Occlusion of a branch of the central retinal vein as a manifestation of hypercoagulability in a patient with lung cancer. A possible paraneoplastic event. Recenti Prog Med.

[10] Asensio-Sánchez VM, Hernaez-Ortega MC, Castresana-Jauregui I (2013). [Central retinal vein occlusion as the first symptom of ovarian cancer]. Arch Soc Esp Oftalmol.

[11] Thomas RH (2001). Hypercoagulability syndromes. Arch Intern Med.

[12] Yust-Katz S, Mandel JJ, Wu J (2015). Venous thromboembolism (VTE) and glioblastoma. J Neurooncol.

[13] Marras LC, Geerts WH, Perry JR (2000). The risk of venous thromboembolism is increased throughout the course of malignant glioma: An evidence-based review. Cancer.

[14] Newton SL, Kalamaha K, Fernandes HD (2018). Temozolomide-induced aplastic anemia treated with eltrombopag and granulocyte colony stimulating factor: A report of a rare complication. Cureus.

[15] Mansour AM, Salti HI, Han DP (2000). Ocular findings in aplastic anemia. Ophthalmologica.

[16] Biousse V, Rucker JC, Vignal C, Crassard I, Katz BJ, Newman NJ (2003). Anemia and papilledema. Am J Ophthalmol.

[17] Hatoum A, Mohammed R, Zakieh O (2019). The unique invasiveness of glioblastoma and possible drug targets on extracellular matrix. Cancer Manag Res.

[18] Galvin R, Sanders MD (1980). Peripheral retinal haemorrhages with papilloedema. Br J Ophthalmol.

[19] Chern S, Magargal LE, Brav SS (1991). Bilateral central retinal vein occlusion as an initial manifestation of pseudotumor cerebri. Ann Ophthalmol.

[20] Voldman A, Durbin B, Nguyen J, Ellis B, Leys M (2017). Fulminant idiopathic intracranial hypertension and venous stasis retinopathy resulting in severe bilateral visual impairment. Eur J Ophthalmol.

